# Not All Immune Checkpoints Are Created Equal

**DOI:** 10.3389/fimmu.2018.01909

**Published:** 2018-08-31

**Authors:** Annika De Sousa Linhares, Judith Leitner, Katharina Grabmeier-Pfistershammer, Peter Steinberger

**Affiliations:** ^1^Division of Immune Receptors and T Cell Activation, Medical University of Vienna, Vienna, Austria; ^2^Division of Clinical and Experimental Immunology, Center for Pathophysiology, Infectiology, and Immunology, Institute of Immunology, Medical University of Vienna, Vienna, Austria

**Keywords:** cancer immunotherapy, inhibitory receptors, immune checkpoints, PD-1, BTLA, LAG-3, CTLA-4

## Abstract

Antibodies that block T cell inhibition via the immune checkpoints CTLA-4 and PD-1 have revolutionized cancer therapy during the last 15 years. T cells express additional inhibitory surface receptors that are considered to have potential as targets in cancer immunotherapy. Antibodies against LAG-3 and TIM-3 are currently clinically tested to evaluate their effectiveness in patients suffering from advanced solid tumors or hematologic malignancies. In addition, blockade of the inhibitory BTLA receptors on human T cells may have potential to unleash T cells to effectively combat cancer cells. Much research on these immune checkpoints has focused on mouse models. The analysis of animals that lack individual inhibitory receptors has shed some light on the role of these molecules in regulating T cells, but also immune responses in general. There are current intensive efforts to gauge the efficacy of antibodies targeting these molecules called immune checkpoint inhibitors alone or in different combinations in preclinical models of cancer. Differences between mouse and human immunology warrant studies on human immune cells to appreciate the potential of individual pathways in enhancing T cell responses. Results from clinical studies are not only highlighting the great benefit of immune checkpoint inhibitors for treating cancer but also yield precious information on their role in regulating T cells and other cells of the immune system. However, despite the clinical relevance of CTLA-4 and PD-1 and the high potential of the emerging immune checkpoints, there are still substantial gaps in our understanding of the biology of these molecules, which might prevent the full realization of their therapeutic potential. This review addresses PD-1, CTLA-4, BTLA, LAG-3, and TIM-3, which are considered major inhibitory immune checkpoints expressed on T cells. It provides summaries of our current conception of the role of these molecules in regulating T cell responses, and discussions about major ambiguities and gaps in our knowledge. We emphasize that each of these molecules harbors unique properties that set it apart from the others. Their distinct functional profiles should be taken into account in therapeutic strategies that aim to exploit these pathways to enhance immune responses to combat cancer.

## Introduction

Although T cells can recognize tumor antigens, they depend on therapeutic intervention to effectively combat malignant cells in cancer patients. While many attempts with antigen-based therapies failed, antigen-independent strategies that enhance T cell responses by blocking inhibitory pathways have been shown to be effective in a significant proportion of treated patients. This therapeutic success is achieved by antibodies often referred to as immune checkpoint inhibitors (ICIs) (1). CTLA-4 was the first immune checkpoint that was targeted to enhance T cell responses in patients suffering from melanoma (2). Antibodies interfering with PD-1 mediated inhibition of T cells and potentially other immune cells were introduced a few years later and have had the greatest success so far (3–6). Inhibitory immune checkpoints help maintaining tolerance and consequently a broad spectrum of side effects–immune-related adverse events (IRAEs)–are observed in treated patients (7). Moreover, monotherapy with ICIs that are currently in use is only beneficial in a subset of cancer patients and frequently leads to acquired resistance (8–10). Consequently, there have been many attempts to evaluate the efficacy of combining PD-1 blockers with conventional cancer treatments (chemotherapy, radiation, surgery) or targeted therapies. Co-administration of PD-1 and CTLA-4 antibodies to patients with melanoma was shown to increase therapeutic efficacy, whereas adverse events were only moderately increased as compared to CTLA-4 blockade alone (11). It is possible that blocking other inhibitory receptors might also augment the therapeutic benefit of PD-1 blockade. Antibodies targeting BTLA, TIM-3, and LAG-3 are promising candidates to boost T cell responses alone or in combination with ICIs disrupting PD-1 mediated inhibition.

Consequently, there is great interest to understand the biology of these inhibitory receptors, which, like CTLA-4, are clearly distinct from PD-1. The original concept of inhibitory receptors was shaped by a group of receptors described on NK cells over 20 years ago (12, 13). These molecules were shown to contain inhibitory motifs (ITIMs), which upon engagement by their ligands, counteract activating signaling processes mediated by ITAM-containing receptors (14). These classical inhibitory receptors exert their function by recruiting SH2-containing phosphatases SHP-1 and SHP-2, which dephosphorylate signaling molecules, thereby directly interfering with activating signaling processes. It was later established that receptors can exert inhibitory functions independent of ITIM motifs. Therefore, inhibitory receptors are now defined by their function rather than by the presence of an ITIM motif in their cytoplasmic domain (13, 15). It is quite clear that BTLA, LAG-3, TIM-3, and CTLA-4 deviate from the classical inhibitory receptors described above. They also differ considerably from PD-1, which can be regarded as the prototypic T cell-expressed immune checkpoint that induces inhibitory intracellular signaling upon engagement with its non-signaling ligands that are preferentially expressed on antigen-presenting cells (APC) and tumor cells.

**Figure 1:**
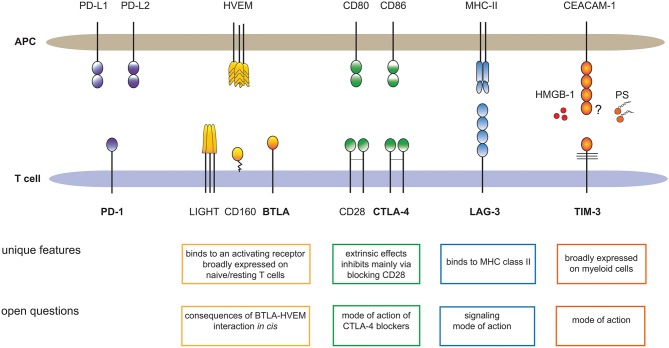
Major T cell expressed immune checkpoints and their ligands. The cartoon summarizes unique features of BTLA, CTLA-4, LAG-3, and TIM-3, which sets these receptors apart from PD-1, the primary immune checkpoint on T cells. In addition, important open questions regarding these pathways are outlined. HMGB-1, high-mobility group box 1; PS, Phosphatidylserine.

Here, we want to illustrate that each of these inhibitory receptors has unique properties and we want to draw attention to important open questions regarding BTLA, LAG-3, TIM-3 and CTLA-4 (Figure 1). Distinct features of each immune checkpoint should be accounted for when developing strategies to exploit these pathways therapeutically and unresolved issues and controversies need to be addressed to better validate their potential as targets in cancer immunotherapy.

## Programmed cell death protein-1 (PD-1)

**Figure 2:**
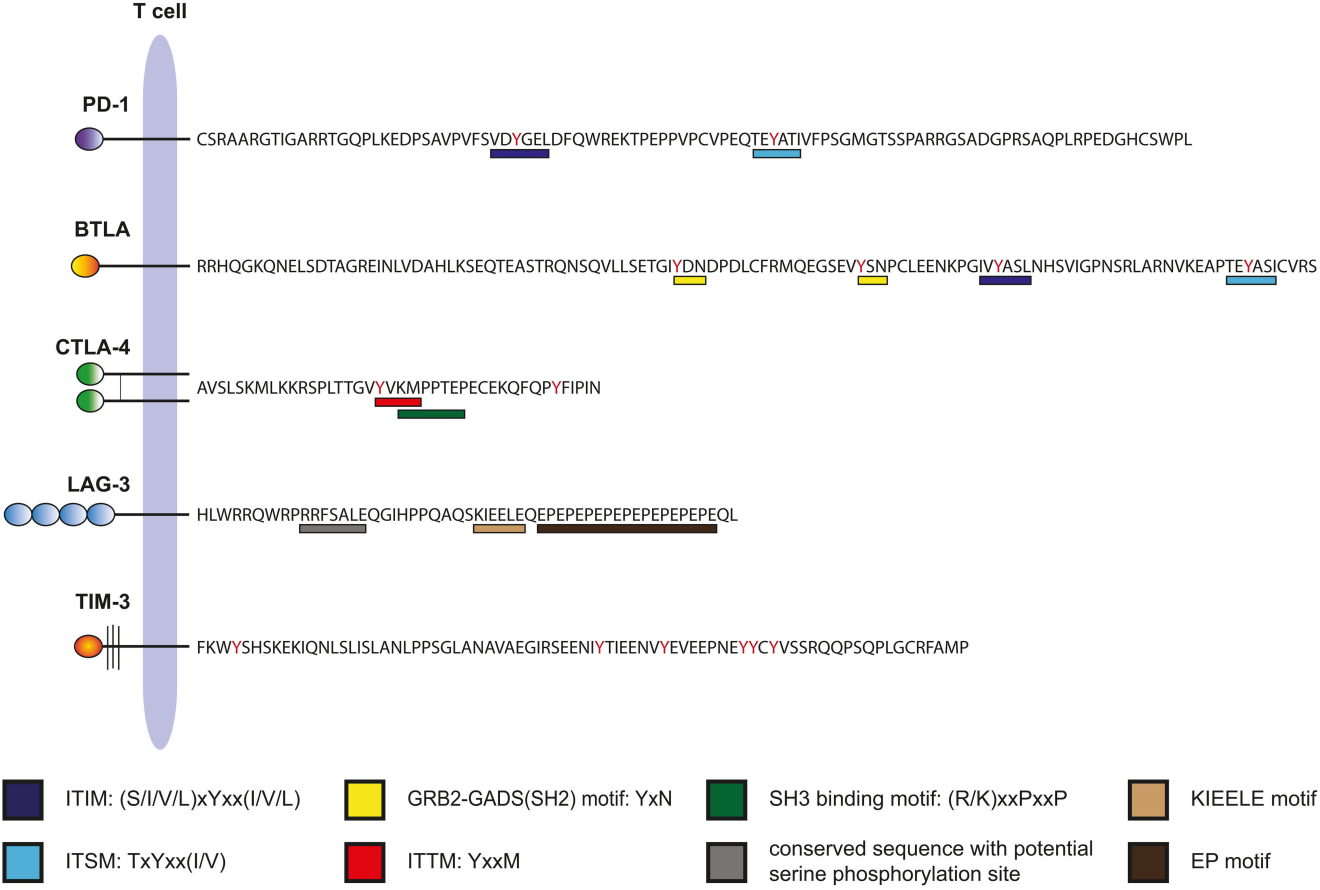
Intracellular domains of human immune checkpoints. The amino acid sequences of the cytoplasmic domains of human PD-1, BTLA, CTLA-4, LAG-3, and TIM-3 are shown. Binding motifs for signaling molecules are indicated.

Programmed cell death-1 (PD-1) is a type 1 transmembrane receptor that belongs to the immunoglobulin superfamily (Ig-SF). Its cytoplasmic domain contains two inhibitory motifs: an immunoreceptor tyrosine-based inhibition motif (ITIM) and an immunoreceptor tyrosine-based switch motif (ITSM) (Figure 2). Following TCR-ligation, the phosphatase SHP-2 associates with the intracellular domain of PD-1 via these motifs (16). However, PD-1 ligation is required for inhibition, suggesting that PD-1 must co-localize with the TCR-CD3 complex or CD28 to exert its function (17). Whereas earlier work has suggested that strong CD28 costimulation can override PD-1 costimulation (18), two recent studies reported that CD28 is a major target of PD-1 signaling (19, 20). The B7-family members programmed cell death ligand-1 (PD-L1) and−2 (PD-L2) are ligands for PD-1. PD-L2 expression is mainly restricted to professional APCs such as dendritic cells (DCs) and macrophages, whereas PD-L1 is broadly expressed on cells of the hematopoietic lineage including activated T cells (21). Inflammatory stimuli induce PD-L1 expression and this ligand is also expressed in a wide variety of non-hematopoietic tissues and importantly in many different types of tumor cells (22, 23). PD-1 is a potent negative regulator of T cell activation and studies on PD-1^−/−^ mice highlighted an essential role of PD-1 in maintaining tolerance and preventing autoimmunity. Mice deficient in PD-1 develop features of a lupus-like disease and autoimmunity is promoted in NOD and MLR mice (24, 25). The interaction of PD-1 with its ligands promotes tolerance and dampens T cell immunity at several levels. PD-1 helps to maintain central tolerance by regulating positive and negative selection (26). It critically contributes to peripheral tolerance, e.g., by promoting Treg induction, expression of PD-ligands on resting DCs and upregulation of PD-L1 on host tissues and endothelial cells during inflammation (27–29). However, PD-1 also limits productive T cell immunity against pathogens and tumor cells (30). PD-1 is induced upon T cell activation, and PD-ligands are constitutively expressed on APCs such as DCs. Consequently, PD-1 is broadly engaged on T cells responding to their cognate antigens. Importantly, PD-1 gains importance on T cells that are exposed to persistent antigenic challenge through antigens derived from chronic viruses or tumor cells. Such T cells enter a state of functional impairment that is often described as exhaustion (31). It was shown that PD-1 is constitutively expressed on mouse, macaque and human CD8 T cells specific for LCMV and HIV antigens, respectively (32–34). Importantly, blockade of PD-1 signaling reverts the functional impairment of exhausted T cells in both models. Signs of exhaustion are frequently observed in tumor resident T cells (35) and their capability to combat tumor cells is frequently impaired by the presence of PD-L1 on their targets. Moreover, blocking PD-1 or PD-L1 was demonstrated to enhance anti-tumor responses in murine models of cancer (36–38). Taken together, these findings provided a rationale for targeting PD-1 to enhance anti-tumor responses in humans. Several antibodies blocking PD-1 signaling by either binding to PD-1 or to PD-L1 have shown clinical efficacy in solid tumors and hematological malignancies such as melanoma, non-small cell lung cancer (NSCLC), renal cell carcinoma (RCC), head, and neck squamous cell carcinoma, cervical cancer, uterine cancer, breast cancer, Merkel cell carcinoma, Hodgkin's lymphoma, diffuse large B cell lymphoma, and follicular lymphoma (39). Although these antibodies represent a great advance in cancer treatment, there is a great variation in patient response to PD-1 blockade with a significant proportion not responding. Consequently, there are intense efforts underway to combine PD-1 blockers with conventional therapies or targeting of other inhibitory receptors to further increase the response rate in cancer patients.

## B and T lymphocyte attenuator (BTLA)

B and T lymphocyte attenuator (BTLA) is a type I transmembrane receptor belonging to the Ig-superfamily. It bears similarities to PD-1; its extracellular domain has an IgV-like fold and its cytoplasmic domain also harbors an ITIM and an ITSM motif, two classical inhibitory motifs (Figure 2). Engagement of BTLA was reported to lead to the recruitment of the SH2-domain containing phosphatases SHP-1 and SHP-2, which subsequently mediate the inhibitory effects of this receptor (40, 41). However, BTLA, which has a long cytoplasmic tail of 111 amino acids can also engage activating signaling pathways via a putative Grb-2 binding motif located upstream of ITIM/ITSM sequences (42) (Figure 2). As implied by its name, BTLA is preferentially expressed on B and T cells, but it is also present on innate immune cells such as monocytes and DCs (43). The only known ligand expressed in human cells is the Herpes virus entry mediator (HVEM), a member of the tumor necrosis factor receptor superfamily (TNFR-SF) (44). HVEM is an activating receptor that also interacts with members of the TNF-SF, LIGHT, and LT-α (43). In addition, HVEM is a binding partner of CD160, which is also a member of the Ig-SF (45). The role of CD160 in T cell activation processes is controversial because both activating and inhibitory effects have been reported (45–47). CD160 is mainly expressed as a GPI-linked molecule and it is currently unclear how this receptor engages the intracellular signaling machinery of T cells.

In mice, BTLA deficiency is associated with hyper-reactive B and T cells and enhanced susceptibility to autoimmunity (43). Interestingly, HVEM-deficiency results in a similar phenotype, indicating that inhibitory BTLA signaling might play a dominant role in the HVEM network (48). Several reports have found that BTLA blockers can enhance human T cell responses when used alone or in combination with antibodies against PD-1 (49–52). Work by Derré and colleagues demonstrated that although BTLA is down-regulated during activation and differentiation, this receptor is prominently expressed on human T cells in the tumor microenvironment and can function to inhibit tumor-specific T cells (53). However, recent studies indicate that the role of BTLA in tumor-resident T cells is complex, as engagement by its ligand HVEM inhibits proliferation and cytokine production but promotes survival of tumor-infiltrating lymphocytes (TILs) (54). Signaling mediated via PI3K recruitment to the Grb-2 binding motif of BTLA has been implicated in these activating effects (54, 55).

### Unique features of BTLA

The intracellular domain of BTLA bears classical inhibitory motifs and it is well established that BTLA mainly functions as a negative regulator of lymphocytes. However, two striking features set BTLA apart from other inhibitory immune checkpoints expressed on T cells. One peculiarity is that it has a ligand that functions as activating receptor. Therefore, the interaction of BTLA with the TNFR-family member HVEM not only generates inhibitory signals in BTLA expressing cells but also stimulatory signals in the cells that express HVEM. Therefore, both BTLA and HVEM have a dual role as a ligand and receptor when they interact with each other (43, 56, 57). It seems counter-intuitive that upon engagement of an inhibitory receptor also an activating signal is generated, and currently the significance of this phenomenon is not understood.

Another unique feature of BTLA is that it is prominently expressed on naïve T cells and tentatively down-regulated upon activation and differentiation (58). This is in stark contrast to other inhibitory immune checkpoints, which are largely absent on naïve cells. PD-1, CTLA-4, or LAG-3 expression is associated with activation and persistent stimulation, which is consistent with a role of these molecules in limiting and terminating immune reactions. Currently the significance of the unusual expression pattern of BTLA expression is not clear.

### Important open questions about BTLA

Elegant work by Cheung and colleagues has shown that BTLA and HVEM interact with each other on cells co-expressing these molecules (59). The majority of human T cells harbor HVEM, and thus BTLA and its ligand HVEM are extensively co-expressed in these cells. It has not yet been addressed whether such *in cis* engagement of BTLA and HVEM during the activation of T cells results in signaling by either of these molecules. However, there is evidence that *in cis* engagement of HVEM prevents the interaction of this receptor with ligands *in trans*, thereby precluding HVEM signaling (59). To date, no studies have addressed whether engagement of BTLA by co-expressed HVEM also attenuates BTLA signaling by interfering with interaction with HVEM *in trans*. The majority of co-inhibitory receptors expressed on T cells is tightly regulated and can only be detected on the surface of activated or “exhausted” T cells. In contrast, BTLA is broadly expressed on T cells and it is tempting to speculate that its function is controlled by co-expression of HVEM. Future studies should test this hypothesis and aim to reveal the interrelationship of BTLA and HVEM on T cells. Eventually, these studies might help to gauge the potential of BTLA as a target of tumor immunotherapy and to devise immune checkpoint inhibitors that optimally target this pathway.

## Cytotoxic T lymphocyte antigen-4 (CTLA-4)

Cytotoxic T cell lymphocyte antigen-4 (CTLA-4) is a type-1 transmembrane protein harboring a IgV-like Ig-domain. Conventional T cells express CTLA-4 upon activation, whereas Tregs express it constitutively (60). CTLA-4 resides in intracellular vesicles and is quickly exported to the surface upon activation. Importantly, CD28, the primary costimulatory receptor, and CTLA-4 share their extracellular ligands CD80 and CD86, but CTLA-4 binds both molecules with higher affinity. Two papers on CTLA-4-deficient mice published in 1995 clearly established that CTLA-4 functions as a negative regulator of T cell responses. These studies demonstrated that mice lacking CTLA-4 suffer from autoimmune phenomena and immune dysregulation which results in early death (61, 62).

The 36 amino acid cytoplasmic tail of CTLA-4 is highly conserved and interaction with several intracellular signaling molecules has been reported (63). Interestingly, CTLA-4 and its activating counterpart CD28 have common intracellular binding partners including the p85 subunit of PI3K and the phosphatase PP2A (63). In Tregs, the protein kinase C-η (PKC-η) associates with CTLA-4 and signaling via the CTLA-4–PKC-η axis was found to be required for contact-dependent suppression (64). Arguably, the best-established relationship of a binding motif within the cytoplasmic tail of CTLA-4 with a function is the YVKM motif that interacts with the clathrin adaptor complex AP-2, thereby promoting internalization and localization of CTLA-4 in intracellular vesicles (Figure 2) (65, 66). For surface expression of CTLA-4 a molecular complex comprised of TRIM, LAX and Rab8 is formed, which shuttle CTLA-4 from the trans-Golgi networt to the surface (67). It has been suggested that the function of the cytoplasmic domain of CTLA-4 is to control the turnover and cellular location of this molecule rather than transmitting inhibitory signals (68).

### Unique features of CTLA-4

Initial research focused on the contribution of the cytoplasmic tail of CTLA-4 to T cell inhibition. These studies revealed that several intracellular signaling molecules can interact with motifs contained in the intracellular domain of CTLA-4. In addition to “classical” effects like recruitment of enzymes that counteract TCR mediated downstream signaling processes (69, 70), it was found that CTLA-4 mediates a reversal of the “stop-signal” initiated upon cognate T cell-APC interaction and thereby prevents efficient cytokine production and proliferation (71). Although these mechanisms contribute to T cell inhibition, there is increasing evidence that CTLA-4 exhibits inhibitory functions that are independent of its intracellular moiety (72–74). Therefore, one unique property of CTLA-4 is that “extrinsic effects,” specifically its capacity to interfere with CD28 costimulation, critically contribute to its function as an attenuator of T cell immunity. Two major mechanisms have been demonstrated in this context. First, CTLA-4, which has a higher affinity for CD80 and CD86 than CD28, binds these ligands and thereby prevents CD28 costimulation (73, 75, 76). More recently, it was shown that CTLA-4 depletes B7 molecules on APC by literally ripping out these costimulatory ligands, a process termed trans-endocytosis (77). However, it is currently not clear to which extend this process contributes to the extrinsic function of CTLA-4. Results of a study were a transgene encoding tail-less CTLA-4 and full length CTLA-4 was introduced into CTLA-4 deficient mice only mice expressing the full length molecule were completely healthy, whereas expression of a tail-less molecule only partly restored immune function. This indicated that both intrinsic and extrinsic effects contribute to maintenance of immune homeostastis by CTLA-4 (78). An important function of CTLA-4 on conventional T cells and Tregs may be the regulation of activating signals via the primary costimulatory receptor CD28. Indeed, induction of autoimmune disease in CTLA-4^−/−^ mice is only observed when *in vivo* CD28 costimulation is in place (79).

### Important open questions about CTLA-4

As outlined above CTLA-4 has been implicated to mediate T cell inhibition by numerous quite distinct mechanisms. Although there is mounting evidence that signaling-independent processes have a major role, the contribution of individual mechanisms is a matter of ongoing debate. Tregs, which have a variety of mechanisms to inhibit immune responses, are characterized by constitutive and high CTLA-4 expression. Studies in mouse tumor models showing that CTLA-4 antibodies can function by depleting intratumoral Tregs via Fc-receptor dependent mechanisms have received much attention (80–82). Recent work by Romano and colleagues demonstrated that patients responding to ipilimumab have higher frequencies of non-classical monocytes and that ipilimumab can mediate killing of CTLA-4^high^ cells by these cells (83). In addition, there is evidence that in melanoma patients response to ipilimumab was associated with the CD16a-V158F high affinity polymorphism (84). Taken together, these results suggest that ipilimumab, which is an IgG_1_ antibody that is fully capable of interacting with Fc-receptors, may mediate killing of Tregs *in vivo*. However, more investigations are required to substantiate that Treg depletion is a major mechanism of ipilimumab action in cancer patients. Such studies might also help determine potential of strategies aiming at Treg depletion in cancer therapy.

## Lymphocyte activation gene-3 (LAG-3)

Lymphocyte activation gene-3 is a type 1 transmembrane protein that has significant homology to CD4 and was first described by Triebel and colleagues in 1990 (85). Expression of LAG-3 has been described on activated T cells, B cells, and NK cells but also on plasmacytoid DCs (85–87). Its extracellular part contains four Ig-like domains and shares high structural homology to CD4. Like CD4, LAG-3 binds to MHC class II molecules, albeit with much higher affinity (88). LAG-3 is heavily glycosylated and interacts with the lectins galectin 3 and the cell surface resident liver sinusoidal endothelial lectin (LSECtin), which is a member of the DC-SIGN family (89, 90).

The 54 amino acid cytoplasmic tail of LAG-3 is devoid of classical motifs involved in the recruitment of inhibitory phosphatases. Instead, it contains a potential serine phosphorylation motif (S454), an unusual sequence consisting of glutamic acid and proline dipeptide motifs (EP motif) and a highly conserved KIEELE motif (Figure 2). A protein termed LAG-3-associated protein (LAP) was shown to bind to the repeated EP motif of LAG-3 but functional effects of this interaction were not studied (91). Follow-up studies on this finding are lacking. The role of the cytoplasmic tail in the function of LAG-3 has to date only been addressed in a singular study by Workman and colleagues published more than 15 years ago (92). The authors expressed wild type and mutated variants of LAG-3 in a murine hen egg lysozyme-specific LAG-3-negative CD4^+^ T cell hybridoma line. They found that wildtype, but not tailless, LAG-3 inhibited IL-2 production in response to antigen. Moreover, the authors reported that LAG-3 inhibition depended on its ligation to MHC class II as well as on the presence of CD4, since a CD4-deficient subline was not inhibited (92). Different LAG-3 mutants were tested and it was found that the KIEELE motif was required, whereas S454 and the EP motif were dispensable for LAG-3 function (92). Collectively, these data suggest that although the presence of CD4 is required for LAG-3 inhibition, this receptor does not simply function by interfering with MHC class II–CD4 interaction, since the intracellular motifs of LAG-3 are required for inhibition. Several studies have shown that LAG-3 functions as an intrinsic negative regulator of CD4^+^ but also CD8^+^ T cells (92–95). In addition, LAG-3 is constitutively expressed on Tregs and can contribute to Treg mediated inhibition (96, 97). Interaction of Treg expressed LAG-3 with MHC class II molecules was shown to induce inhibitory signaling pathways in DCs (98). A number of studies in murine tumor models have provided a rationale for LAG-3 blockade to limit tumor growth. It was shown that LAG-3 antibodies alone or in combination with PD-1 blockers curtailed growth of malignant cells and promoted tumor clearance (99–102). Several antibodies targeting LAG-3, including the bispecific agent MGD013 that simultaneously binds LAG-3 and PD-1, are in clinical development. Most of these aim at enhancing T cell responses, but a depleting antibody that should function by killing activated effector memory T cells, thus reducing unwanted T cell responses, is also being developed (103, 104). In addition, IMP321, a LAG-3 immunoglobulin fusion protein that exerts immune potentiating functions by activating APCs via MHC class II molecules, is being tested in several clinical trials (103, 105).

### Unique features of LAG-3

A striking feature of LAG-3 is that it ligates to MHC class II molecules rather than to a generic co-inhibitory ligand. Related to this, LAG-3 has a large extracellular domain compared to other T cell expressed co-inhibitory molecules like PD-1, BTLA, and CTLA-4. In addition, LAG-3 has an unusual cytoplasmic tail containing motifs that are not found in other co-inhibitory receptors. Therefore, inhibitory mechanisms of LAG-3 are likely to be unique and clearly distinct from those exerted by other immune checkpoints; potential extrinsic effects will affect the antigen-specific signals rather than costimulatory signals (signal 1 rather than signal 2) and intrinsic effects will engage unique pathways that are not used by other inhibitory receptors.

### Important open questions about LAG-3

The mode of action of LAG-3 mediated inhibition is currently incompletely understood. LAP binds to the EP motifs of LAG-3 but the consequences of this interaction are not known (91). The KIEELE domain is highly conserved and was described to be required for the inhibitory function of LAG-3. However, there is a complete lack of data showing how the intracellular signaling machinery of T cells connects with this motif to counteract activating T cell signaling processes. Workman and colleagues have described that LAG-3 inhibits CD4-dependent, but not CD4-independent T cell function (92). Thus, it is possible that the role of the intracellular domain of LAG-3 is to promote the extrinsic effects of LAG-3 for instance by ensuring optimal spatial orientation of LAG-3 in the immunological synapse. However, direct inhibition of CD8^+^ T cells by LAG-3 has also been described and distinct mechanisms have to be involved for such a function of LAG-3 (93–95). We have found that blocking LAG-3 alone or in combination with PD-1 on T cells stimulated with allogeneic DC or virus antigens had limited efficacy (49, 50). In general, there are scarce data describing a robust effect of LAG-3 on human T cell responses *in vitro*. Establishing stimulation conditions for primary human T cells where LAG-3 blockade exerts a strong and reproducible effect would be valuable to further our understanding of LAG-3 function and aid the development of improved therapeutic strategies targeting this immune checkpoint in T cells.

Another important issue is the consequence of MHC class II engagement by LAG-3. The LAG-3 fusion protein IMP321 shows adjuvant properties and enhances immunogenicity of tumor vaccines (105). Induction of DC maturation via engagement of MHC class II has been proposed as a mechanism underlying this effect (106, 107). Interestingly, binding of MHC class II molecules on a CD4 T cell clone by a LAG-3 fusion protein inhibited proliferation and cytokine production upon stimulation with antigen (108). Moreover, Tregs were shown to inhibit DC maturation via LAG-3 (98). Thus it appears that engagement of MHC class II molecules by membrane-bound or soluble LAG-3 can transduce either activating or inhibitory signals and dissecting the mechanisms behind this functional dichotomy will certainly help to understand the complex pathways used by LAG-3 to regulate immune responses. LAG-3 is also released from CD4 T cells after activation but it is not known whether this has a role in immune regulation (109).

## T cell immunoglobulin and mucin-domain containing protein-3 (TIM-3)

T cell immunoglobulin and mucin-domain containing protein-3 (TIM-3) is a member of the TIM family, which has two additional members in humans: TIM-1 and TIM-4. TIM-molecules are type I transmembrane proteins that contain an N-terminal IgV-like domain and a mucin domain (110). TIM-3 is constitutively expressed on innate immune cells such as monocytes/macrophages, DCs, mast cells, and mature NK cells, whereas on T cells its expression is associated with activated and terminally differentiated states (110–113). Several ligands have been proposed for TIM-3. Like all TIMs, it binds phosphatidylserine (PtdSer), yet compared to TIM-1 and TIM-4, its capacity to interact with these molecules appears to be considerably lower (110, 114). TIM-3 also binds to high-mobility group box 1 (HMGB-1), a damage-associated molecular pattern protein that is released from stressed innate immune cells and can interact with different molecules including nucleic acids and lipopolysaccharide (LPS) (115). Based on intracellular binding experiments Galectin-9 (Gal-9) was reported to serve as a binding partner for TIM-3 (116). The galectins are a family of beta-galactoside-binding proteins and Gal-9 was also implicated in binding 4-1BB, CD40, CD44, and Dectin-1 (117–120). We performed a series of experiments that produced no evidence for a specific interaction of human or mouse TIM-3 with Gal-9 (121). CEACAM-1, a co-inhibitory molecule expressed on T cells that functions as a self-ligand, was reported as another ligand for TIM-3 (122). An interaction between TIM-3 and CEACAM-1 on cell surfaces was not shown in this study. Instead, co-precipitation experiments were performed and the crystal structure of a heterodimer of the V-domains of human CEACAM-1 and human TIM-3 was published (122). The heterodimer models have since been withdrawn and further work is required to establish an interaction between CEACAM-1 and TIM-3 (123).

Human TIM-3 has a cytoplasmic tail of 71 amino acids that lacks classical activating or inhibitory signaling motifs like ITAMs or ITIMs (Figure 2). However, several studies report that the cytoplasmic domain of TIM-3 can mediate intracellular signaling in T cells and myeloid cells. Two tyrosines (Y256 and Y263 in human TIM-3) whose phosphorylation enables interaction with SH2 domain containing molecules appear to have a significant role in this process. Intracellular signaling proteins that have been reported to interact with TIM-3 include p85 of PI3K, PLC-γ, ZAP-70, Lck, and SLP-76 (124). Rangachari et al. found that HLA-B-associated transcript 3 (Bat3) associates with the cytoplasmic domain of TIM-3, thereby preventing T cell dysfunction and exhaustion (125). Recent work by Avery and colleagues showed that TIM-3 promotes Akt/mTOR signaling and is essential for optimal effector T cell responses (126).

An autoimmune phenotype of mice lacking TIM-3 was not described but consistent for an inhibitory role of TIM-3 in immunity these animals were found to be refractory to tolerance induction (127). Gorman et al. however found that TIM-3 knockout mice had reduced magnitudes of both primary and secondary CD8 T cell responses. They showed that this effect was cell intrinsic, suggesting that TIM-3 can mediate a stimulatory effect on CD8 T cell responses (128).

### Unique features of TIM-3

Unlike the other immune checkpoints described in this review, TIM-3 is constitutively expressed on several cell types of the myeloid lineage. TIM-3 acts as a receptor for ligands like HMGB-1 and phosphatidylserine, which is consistent with a molecule that is primarily expressed on innate immune cells. CTLA-4, PD-1, LAG-3, and BTLA interact with cell surface molecules preferentially expressed on professional APCs, whereas APC-expressed membrane-bound ligands for TIM-3 have not been reported. Although TIM-3 is present on activated and exhausted T cells, a recent study reported that TIM-3-positive cells in breast cancer cell samples were of myeloid rather than T cell origin (129). Thus, therapeutic approaches targeting TIM-3 are likely to have a strong impact on APCs such as macrophages and DCs.

### Important open questions about TIM-3

TIM-3 is expressed in many immune cells and activating as well as inhibitory functions have been ascribed to this receptor. Phosphatidylserine, HMGB-1, Galectin-9, and CEACAM-1 were proposed as binding partners for this molecule, but it is currently not clear whether all of these molecules act as *bona fide* TIM-3 ligands. In many studies, TIM-3 function was not linked to a specific TIM-3 ligand, and Galectin-9 and CEACAM-1 can regulate T cells independent of TIM-3 (120, 130–133).

Several reports found that antibodies against human TIM-3 enhance T cells responses alone or in combination with PD-1 blockers and thus provide a rationale to explore strategies to enhance anti-cancer immunity by targeting TIM-3 (49, 50, 113, 134, 135). TIM-3 antibodies could directly act on T cells or indirectly by potentiating APC functions, which in turn could enhance T cell responses. In this context, it should be noted that TIM-3 antibodies were shown to induce activating signals in human DCs (5, 111). Gain of function studies on TIM-3 in human T cell lines have yielded conflicting results; while one group obtained results that point to an activating role of TIM-3 (124), others have observed effects that are consistent with an inhibitory role of TIM-3 (136). T cell reporter systems based on the human T cell line Jurkat are powerful tools to assess mechanisms of co-inhibition and to test immune checkpoint inhibitors. Although such reductionist assay systems for evaluating antibodies against PD-1, CTLA-4, BTLA, and LAG-3 are commercially available and have been described in the literature (72, 137–140), a validated test system for antibodies targeting TIM-3 has not yet been described to our knowledge. A recent report by Sabins and colleagues demonstrated that a TIM-3 antibody that was used in several studies to target human TIM-3 could function as an agonist and promoted CD8 T cell differentiation through activation of mTORC1 (141). Thus, it will be necessary to address whether functionally active antibodies to human TIM-3 act as agonists or antagonists to understand the role of TIM-3 in human T cell responses.

## General open questions and outlook

### Exhaustion and immune checkpoints

It is generally accepted that persistent stimulation with an antigen can result in a state of functional impairment referred to as exhaustion in T cells specific for virus and tumor antigens. A landmark paper by Blackburn and colleagues showed that exhausted T cells can upregulate several co-inhibitory receptors (142). Subsequently, it was shown that Melan-A-specific T cells in patients with melanoma resemble exhausted T cells in chronic infections (143). Importantly, several studies have demonstrated that PD-1 antagonists can revert dysfunction in exhausted T cells (32, 33, 144, 145). Based on these findings, immune checkpoint receptors have been phenotypically and functionally linked to T cell exhaustion (146, 147). Consequently, it is often inferred that immune checkpoint inhibitors mainly function to reinvigorate exhausted T cells. Although inhibitory receptors are involved in T cell exhaustion, it is important to emphasize that the expression of immune checkpoint inhibitors on T cells is by no means limited to exhausted populations (147, 148). In addition, the presence of a particular inhibitory receptor on exhausted T cells neither proves that the receptor is the cause of their state nor that it critically contributes to their functional impairment (13). A better understanding on the relationship of inhibitory immune checkpoints and their role in exhaustion is highly desired and will help to understand the potential but also the limitations of ICIs in targeting exhausted tumor specific T cells.

### Tregs and inhibitory immune checkpoints

Tregs and T cell-expressed inhibitory immune checkpoints play important roles in maintaining peripheral tolerance. However, they can both limit protective immunity against pathogens and tumor cells. As summarized in a recent review addressing the immune checkpoint inhibitors in Tregs, these cells constitutively express immune checkpoints like CTLA-4 but also PD-1, BTLA, LAG-3, and TIM-3, and upregulate inhibitory receptors during activation and at tumor sites (1). Although there is ample evidence for a role of co-inhibitory receptors in Treg function (96, 149, 150), many aspects of the interrelation between these two pillars of tolerance are incompletely understood. Specifically, it is not clear how immune checkpoints that inhibit T cells by downregulating intracellular signaling pathways function in Tregs: is engagement of such receptors on Tregs mainly attenuating or enhancing their regulatory function? If the former would be true, ICI-therapy would potentiate Treg function, which could result in reduced efficacy of such regimens. Whereas in the latter case, immune checkpoint inhibitors might exert their beneficial function at least in part by targeting Tregs.

### Emerging immune checkpoints

There are several additional co-inhibitory pathways that are implicated in limiting T cell responses and thus might have potential in cancer immunotherapy. One such protein is TIGIT (T cell immunoreceptor with Ig and ITIM domains), which bears similarities to CTLA-4 as it shares binding partners with an activating receptor (CD226), which binds these ligands with lower affinity (151–154).

V-domain Ig suppressor of T cell activation (VISTA), also known as B7-H5, PD-1H, and Gi24, is expressed on T cells, myeloid cells, and NK-cells (155). VISTA, an orphan receptor on T cells, can also function as a ligand for an unknown receptor on T cells (156–158). Studies in mice and murine cells indicate that VISTA has an inhibitory role in immunity. VISTA was knocked out in mice by two independent approaches and both showed signs of enhanced immune activity and autoimmunity albeit to different degrees (159, 160). In addition, there are several studies in mice that suggest that blocking VISTA might enhance tumor immunity (155, 156, 158, 161, 162). In addition several studies show VISTA expression in tumors and treatment with ipilimumab was found to upregulate VISTA in patients with prostate cancer (162–165). Therefore, antibodies blocking VISTA on T cells as well as the interaction of APC-expressed VISTA with its unknown receptor expressed on T cells may have potential in cancer immunotherapy since they could enhance T cell responses by disrupting two inhibitory signaling pathways in T cells.

Surprisingly few studies have addressed the role of VISTA in human T cells and myeloid cells. Lines et al. reported that a VISTA immunoglobulin fusion protein blocks T cell activation and promotes the generation of human Tregs (166). By contrast Baraj and colleagues found that overexpression of VISTA on human monocytes promoted their activation and subsequently enhanced T cell responses (167). To date there is a lack of information not only regarding receptors and ligands on T cells and APC, respectively, that mediate the proposed effects of VISTA but also on downstream signaling events induced upon VISTA engagement. Despite, this and based on promising result in mice, a clinical trial with a monoclonal antibody to VISTA was initiated (NCT02671955).

B7-H7, also known as HERV-H LTR associating 2 (HHLA2), is a member of the extended B7 family and inhibits proliferation and cytokine production of human CD4 and CD8 T cells (168). B7-H7 is expressed on human APCs such as monocytes or B cells, but it is also widely expressed in non-hematopoietic tissues and cancers (168, 169). CD28H, also known as TMIGD2, was shown to function as a binding partner for HHLA2 (170). HHLA2 was designated as B7-H5 in this publication and it thus should be stressed that HHLA2 is distinct from VISTA, which has also been referred to as B7-H5. The interaction of TMIGD2 with HHLA2 has since been confirmed by an independent study (169). Interestingly, engagement of TMIGD2 was found to costimulate cytokine production and proliferation in human T cells (170). Thus, it is possible that HHLA2 interacts with another yet-unidentified inhibitory receptor on T cells. Interestingly, neither HHLA2 nor TMIGD2 are expressed in mice and rats (168, 170).

In addition, there are orphan ligand molecules including B7-H3 (CD276), B7-H4 (also known as B7S1, B7x or VTCN1), and ILDR2 that have been reported to inhibit T cell responses (171–177). The identification of receptors for orphan ligands like B7-H3 will be mandatory to target pathways involving these molecules to enhance T cell responses (178). B7-H3 is broadly expressed in cancer cells and B7-H3 antibodies targeting B7-H3^+^ tumors are currently being tested in several clinical trials (179). It is currently not known whether these B7-H3 antibodies interfere with T cell inhibitory effects of this molecule.

### The future: novel immune checkpoint inhibitors and beyond

The arrival of ICIs has dramatically changed the therapeutic landscape of cancer. Despite the enormous success of these immunotherapies, it is becoming increasingly clear that combining ICIs with a second drug may have superior potential to combat cancer. Currently, numerous clinical trials testing combinations of established immune checkpoint inhibitors (PD-1 and PD-L1 antibodies) with conventional treatments (chemotherapy, radiation, or targeted therapy) are carried out and are likely to result in improved treatment modalities for many different types of cancer (180). It is noteworthy to mentioned that ICIs are not the only mean to target inhibitory receptors. Taylor et al. have recently shown that glycogen synthase kinase 3 (GSK-3) has a key role in the regulation of PD-1 expression in CD8^+^T cells (181). Follow up work by the same group has demonstrated that GSK-inhibitors are as effective in enhancing anti-tumor responses in preclinical models as PD-1 and PD-L1 antibodies (182).

Promising clinical data were obtained upon co-administration of antibodies targeting PD-1 and CTLA-4 (11, 183). These two immune checkpoints might mediate anti-tumor effects through distinct non-redundant mechanisms (184, 185). The CTLA-4 antibody ipilimumab acts early during T cell activation and mainly exerts extrinsic effects by outcompeting the primary costimulatory receptor CD28. It promotes the expansion of Th1-like CD4 T cells and potentially the deletion of tumor-resident Tregs. In contrast, PD-1 blockade mainly acts intrinsically on tumor-infiltrating exhausted-like CD8 T cells (184). These cells expand but maintain PD-1 expression indicating that PD-1 blockade does not reprogram them into a non-exhausted state, which is consistent with a epigenetic regulation of exhaustion (184). In addition, these results suggests that despite their exhausted like phenotype these cells are capable to expert potent anti-tumor activity following PD-1 blockade. Since PD-L1 is frequently expressed on tumor cells, PD-1 blockade can have a dual role in the tumor microenvironment–expansion of effector cells and also promoting anti-tumor effector functions.

The successful co-targeting of PD-1 and CTLA-4 and encouraging results obtained in preclinical models that combined PD-1 antibodies with other immune checkpoint inhibitors has fostered strategies to combine immune checkpoint inhibitors to enhance anti-tumor responses in patients with cancer. Several clinical trials where PD-1 antibodies are tested in combination with antibodies targeting TIM-3 and LAG-3 are ongoing (https://clinicaltrials.gov). Distinct properties of PD-1 versus TIM-3 and LAG-3 might result in synergistic effects of such combinations.

Adoptive therapy with T cells genetically engineered to express chimeric antigen receptors (CARs) or TCRs specific for tumor antigens is able to induce impressive anti-tumor responses. However, the upregulation of inhibitory receptors in genetically engineered T cells following transfer reduces their efficacy (186, 187). Multiple clinical trials investigating combinations of CAR T cells with antibodies to PD-1 or PD-L1 are ongoing (188). Engineering CAR T cells or TCR-transgenic T cells that are refractory to inhibition by immune checkpoints represents a promising future avenue to specifically protect engineered tumor-specific T cells against functional impairment through inhibitory pathways such as PD-1/PD-L1. This could be achieved by silencing or knocking out inhibitory receptors but also by co-introducing genes encoding PD-1/PD-L1 antibodies or so-called chimeric switch receptors (188–190).

The triumph of immune checkpoint inhibitors has underpinned that T cells have the potential to efficiently fight tumor cells, but in the majority of cases can only do so upon therapeutic intervention. Immune checkpoint blockade is effective but associated with severe side effects since it interferes with vital mechanisms of peripheral tolerance. Recent work by the Schreiber group has identified T cells that are reactivated upon immune checkpoint inhibitor treatment and mediate tumor rejection in a mouse model. The authors went on to show that tumor rejection can also be achieved by specifically boosting these T cells by peptide vaccination (191). The identification of antigens that are recognized by T cells in patients responding to immune checkpoint therapy might thus offer possibilities to target these antigens by vaccination or introduction of TCR-transgenic autologous T cells. Such approaches may increase the specificity of tumor targeting, thereby potentially enhancing therapy effects while reducing autoimmune toxicity.

Specificity is a hallmark of adaptive immunity and it seems paradoxical that immune checkpoint inhibition, which is an antigen-independent approach, has had the most spectacular success in cancer immunotherapy to date. Recent technological progress has facilitated the identification of mutations, which give rise to neoantigens in the tumors of individual cancer patients (192, 193). Studies in mouse models have demonstrated that vaccination with neoantigens can result in tumor control (191, 194). Strategies that combine patient-tailored approaches aimed at enhancing immune responses to individual neoantigens (e.g., by synthetic vaccines, oncolytic viruses or tumor radiation therapy but also adoptive therapy with *in vitro* expanded neoantigen-specific T cells) and interference with inhibitory pathways might represent particular promising avenues to improve anti-cancer immunotherapy (195, 196).

## Author contributions

AD, JL, KG-P, and PS wrote the manuscript. AD and JL prepared figures.

### Conflict of interest statement

The authors declare that the research was conducted in the absence of any commercial or financial relationships that could be construed as a potential conflict of interest.
